# Comparative effects of exercise training on serve performance and upper-body power in tennis players: a systematic review and network meta-analysis

**DOI:** 10.3389/fphys.2026.1920673

**Published:** 2026-08-28

**Authors:** Song Hu, Fengjiao Wang, Jia Liu, Yong Zhi, Guoyu Wang

**Affiliations:** 1 Graduate School of Physical Education, Pukyong National University, Busan, Republic of Korea; 2 School of Health Sciences, Universiti Sains Malaysia, Kelantan, Malaysia; 3 College of Sports Science, Jilin Sport University, Changchun, China; 4 Department of Industrial Design, Pukyong National University, Busan, Republic of Korea

**Keywords:** exercise training, network meta-analysis, serve accuracy, serve velocity, tennis, upper-body power

## Abstract

**Objective:**

To systematically compare the effects of different exercise training interventions on the serve performance and upper-body power of tennis players.

**Methods:**

PubMed, Web of Science, Cochrane Library, Embase, CNKI, WanFang Data and VIP database were systematically searched from inception to May 25, 2026. Healthy tennis players with an intervention period ≥4 weeks were included. Standardized mean difference (SMD) and 95% confidence interval (CI) were used as effect sizes. Pairwise meta-analysis and network meta-analysis were conducted on Stata 16.0, and the different training interventions were sorted using the surface under the cumulative ranking curve (SUCRA). The quality of each included study was evaluated using Cochrane’s Risk-of-Bias Assessment Tool, and the evidence quality of network meta-analysis was assessed using CINeMA.

**Results:**

Totally 20 studies involving 606 tennis players were included. Network meta-analysis shows that plyometric training (PT, SMD = 0.91, 95%CI: 0.56 to 1.26), resistance training (RT, SMD = 0.85, 95%CI: 0.46 to 1.23), core stability training (CST, SMD = 0.82, 95%CI: 0.24 to 1.41) and multi-modal training (MMT, SMD = 0.77, 95%CI: 0.33 to 1.20) can all significantly increase serve velocity compared with regular training (REG). The SUCRA ranking shows that PT ranks first in terms of serve velocity (72.6%). There is no significant difference between each training intervention and REG in terms of serve accuracy or upper-body power. CST is ranked first by SUCRA for both indicators (serve accuracy: 75.3%; upper-body power: 81.9%). The CINeMA shows that the quality of evidence is high or moderate for some comparison results in terms of serve velocity, but is generally low for serve accuracy and upper-body power.

**Conclusions:**

All types of exercise training interventions can effectively improve the serve velocity of tennis players, among which PT has the best effect. CST shows potential advantages in serve accuracy and upper-body power, but the quality of evidence is weak. PT may be considered the main training method to improve serve velocity, particularly in young tennis players, and should be combined with serve technique and placement control training. CST can be used as an auxiliary intervention for serve accuracy training.

## 1 Introduction

Tennis serve performance is one key factor that determines the result of the play (Cavedon et al., 2014), because serve is used frequently in tennis and has a decisive impact on the score (Bonato et al., 2015). Indicators such as the first-serve scoring rate and ACEs are closely related to the final victory (Fitzpatrick et al., 2024). A higher quality of serve means the player can take more initiative in the point (Prieto-Lage et al., 2023). Especially when the first serve is successful and a short rally occurs, the server can take the initiative and win the point (O’Donoghue and Brown, 2008). The quality of serve is affected by many factors, such as velocity, rotation and accuracy. Serve velocity is the key factor affecting the tennis player’s serve performance, and is affected by various factors, such as movement technique, body structure and physical fitness. Among them, the improvement of upper-body power seems to positively impact serve velocity (Colomar et al., 2022). An effective way to improve serve performance is to maintain accuracy while increasing velocity (Keller et al., 2021). Although a faster serve velocity can enhance the offensiveness of serve, it can be hardly converted into a score advantage if there is no precise placement control. An analysis of Wimbledon Tennis championships shows that high-level players can maintain high serve velocity and relatively stable accuracy during long matches (Maquirriain et al., 2016). Therefore, simultaneously maintaining serve velocity and serve accuracy is the key to improving the serve level.

Since tennis is a high-intensity intermittent sport (Groppel and Roetert, 1992), players have to complete quick start, braking, change of direction, and strokes in rallies (Qin et al., 2026). Thus, good strength, velocity, and aerobic and anaerobic metabolic capabilities are important physiological foundations for maintaining exercise performance (Guo et al., 2024). Especially during serving, the player has to complete rapid force output in a short time and maintain good neuromuscular function and physical fitness during repeated serves (Zhou et al., 2025). Exercise training can directly improve neuromuscular function and fatigue resistance, and thus provides a basis for improving serve performance compared with the intervention of emotion regulation, attention control or game strategy (Kovacs, 2006). Reportedly, 8 weeks of plyometric training increased the average serve velocity of tennis players by 3.78%, but did not improve serve accuracy (Behringer et al., 2013). Moreover, 6 weeks of multi-modal training including core strength, elastic band resistance and medicine ball exercises significantly increased serve velocity, but did not obviously impact serve accuracy (Fernandez-Fernandez et al., 2013). Lambrich and Muehlbauer (2022) quantified the impact of exercise training on the physical fitness and stroking velocity of healthy tennis players through a meta-analysis. The improvement effect of exercise training was significant on agility (SMD = 0.93), balance ability (SMD = 0.88), and stroking velocity (SMD = 0.90), and moderate on upper-body power (SMD = 0.72) (Lambrich and Muehlbauer, 2022). Another meta-analysis shows that both resistance training (effect size = 0.53; small effect) and multi-modal training (effect size = 0.79; medium effect) can significantly increase serve velocity, but core training has no significant effect (effect size = 0.32) (Deng et al., 2024).

Previous research shows that exercise training may positively impact tennis players’ serve performance and related physical fitness indicators. However, the intervention effects of different training methods are not completely consistent, and the existing evidence is mainly based on pairwise meta-analysis (PMA), which usually can only compare the difference between a single training intervention and a control condition. Due to the lack of direct and indirect comparisons of multiple training interventions, the relative effectiveness and ranking of the pros and cons of different exercise training interventions in improving serve velocity, serve accuracy and upper-body power are still unclear. On this basis, network meta-analysis (NMA) is needed to comprehensively compare and rank the effects of different exercise training interventions, so as to provide an evidence-based basis for formulating training programs for tennis players.

## 2 Methods

This systematic review and NMA protocol has been registered with PROSPERO (registration number: CRD420261404731) and follows the PRISMA 2020 guidelines and the extended statement for NMA (PRISMA-NMA) for reporting.

### 2.1 Search strategy

We searched seven databases, including four English databases (PubMed, Web of Science, Cochrane Library, Embase) and three Chinese databases (CNKI, WanFang Data, VIP Database). The search time ranged from inception to May 25, 2026. We also searched the reference lists of relevant reviews to ensure a comprehensive search. The search strategy is elaborated in **Supplementary Table 1**.

### 2.2 Inclusion and exclusion criteria

The inclusion and exclusion criteria were formulated based on the participants, intervention, comparison, outcomes, and study design (PICOS) framework. The specific criteria are shown in **Table 1**.

**Table 1:** Inclusion and exclusion criteria based on PICOS framework.

Elements of PICOS	Inclusion criteria	Exclusion criteria
P (Participants)	Healthy tennis players	Injured, or sick tennis players
I (Intervention)	Exercise training intervention. Long-term periodized training (≥4 weeks)	Non-exercise training intervention. Acute experiment (pre-game/pre-training warm-up training only)
C (Comparison)	Regular training or no intervention	No control group or active controlled study
O (Outcomes)	Indicators related to serve performance or upper-body power	No relevant outcome indicators reported
S (Study design)	Parallel control design	Single-group before-and-after studies, reviews, dissertations, conference abstracts, etc.

#### 2.2.1 Inclusion criteria

The inclusion criteria are as follows:

1. Participants: healthy tennis players, regardless of gender or age. Competitive levels include (1) competitive-level players: players aged 18 years or older who participate in competitive events or college teams; (2) young players: players under 18 years old who have regular tennis training or competition experience. Studies targeting beginner, amateur, or recreational players were excluded.
2. Intervention: To include long-term periodic exercise training interventions, (1) training period ≥ 4 weeks, (2) clear training period arrangement, and (3) training goal to improve exercise performance must be met at the same time. Only interventions focusing on physical training were included, and non-physical training interventions focusing on visual, cognitive or psychological skills training were excluded.
3. Comparison: A control group receiving regular tennis training, regular physical training, regular technical and tactical training, or no intervention was included.
4. Outcomes: Indicators related to serve performance or upper-body power.
5. Study design: parallel-controlled design.

#### 2.2.2 Exclusion criteria

The exclusion criteria are as follows:

1. Participants: Tennis players who suffer from injuries, illness or other health problems that may affect their performance.
2. Interventions: (1) acute experimental intervention (e.g., warm-up training before competition, or training only); (2) non-exercise training intervention (e.g., non-physical training intervention focusing on visual, cognitive or psychological skill training); (3) mixed intervention that combines exercise training with other interventions (e.g., neuromuscular electrical stimulation, drug intervention or special diet control).
3. Comparison: Lack of control groups or active controls.
4. Outcomes: not reporting outcome measures related to serve performance or upper-limb power.
5. Study design: single-group before-and-after study, review, dissertation, conference abstract, and other studies.

### 2.3 Intervention types and outcome indicators

#### 2.3.1 Types of interventions

The types and definitions of exercise training interventions involved in this study are shown in **Table 2**.

**Table 2:** Types and definitions of exercise training interventions.

Type of intervention	Definition
Regular training (REG)	Training conditions in which only regular training, traditional physical training or technical and tactical training was carried out and no additional special intervention was received.
Resistance training (RT)	The main purpose is to improve muscle strength or shoulder joint rotator strength by resisting external resistance. It mainly includes resistance training with equipment, free weights, elastic bands, dumbbells and other strength training.
Plyometric training (PT)	The main purpose is to improve explosive power, rapid strength or stretch-shortening cycle ability, and is achieved through rapid eccentric contraction followed by explosive concentric contraction. It mainly includes jumping training, medicine ball throwing training, and rapid telescopic compound movements.
Core stability training (CST)	The main purpose is to strengthen the muscle strength and stability of the trunk, pelvis and hip areas. The training actions mainly include plank support, side bridges, abdominal crunches, and supine leg raises.
Multi-modal training (MMT)	A compound intervention consists of two or more specialized training methods at the same time. The training content is rich and diverse and is not limited to a single training method. e.g., a combination of core training, resistance training, and medicine ball training.

#### 2.3.2 Outcome indicators

The main outcome indicators are related to serve performance, including serve velocity (e.g., peak serve velocity, average serve velocity) and serve accuracy (e.g., placement accuracy, serve success rate).

The secondary outcome indicators are related to upper-body power assessed through medicine ball throwing, including test scores of medicine ball single-hand forward throwing, and double-hand overhead throwing.

### 2.4 Study selection and data extraction

Literature was screened on EndNote 21. Duplicate literature was first removed. Then two researchers independently conducted preliminary screening of titles and abstracts to exclude studies that clearly did not meet the criteria. Studies that may meet the criteria were further evaluated by reading the full texts to determine the final included literature. Disagreement was solved through discussion with a third researcher.

Data were extracted from the final included studies. The extracted contents include 1) Research background: author, year; 2) Player characteristics: average age, sample size, player experience and competitive level; 3) Intervention plan: training type, training cycle, training frequency and single training duration; 4) Bias risk evaluation indicators; 5) Relevant outcome indicators and outcome measurements of concern. When the data were presented in the chart form, the chart data were extracted on Web Plot Digitizer 5.0 (https://automeris.io). Specifically, two researchers conducted cross-checking. Any disagreement was discussed with a third researcher to reach a consensus.

### 2.5 Risk of bias assessment

Two researchers independently used the ROB assessment tool recommended by Cochrane Handbook 5.1.0 to evaluate the ROB of the included studies, and cross-checked the assessment results. The assessments include 1) random sequence generation; 2) allocation concealment; 3) blinding of participants and intervention implementers; 4) blinding of outcome assessors; 5) completeness of data; 6) selective reporting; 7) other sources of bias. The result of each item was low, unclear, or high risk of bias. The results of the ROB assessments were visualized on Review Manager 5.4.

### 2.6 Data analysis

This study was based on the frequentist framework. PMA and NMA were conducted on Stata 16.0. Since the measurement methods and measurement units of the outcome indicators were not completely consistent among the included studies, the standardized mean difference (SMD) was used as the effect size for merging, and its 95% confidence interval (CI) was calculated. Specifically, Hedges’ g was used to estimate the SMD to reduce the potential bias in effect size estimation in small sample studies. Given that the included studies differ in players’ age, competitive level, training experience, and outcome indicator measurement methods, we uniformly used a random effects model to merge effect sizes.

To reduce the impact of baseline differences on intervention effect estimates, we used the change score before and after intervention to merge effect sizes. The mean change was obtained by subtracting the pre-intervention mean from the post-intervention mean. When a study did not report the standard deviation of change, it was calculated according to the formula recommended by the Cochrane Handbook:


$$SD_{change}=\sqrt{S{D_{pre}}^{2}+SD_{post}^{2}-2R\times SD_{pre}\times SD_{post}}$$

where R is the correlation coefficient between the measured values before and after the intervention. When an original study did not report the correlation coefficient, we referred to the recommendations of the Cochrane Handbook and related methodological literature and set R = 0.5 for estimation (Chi et al., 2023; Veronique and Neil, 2013).

In NMA, when there was a closed loop in the evidence network, the global consistency test was first performed. If there was no statistical difference in the test results (P>0.05), the consistency model was used; otherwise, the inconsistency model was used. Local consistency was evaluated using the node-splitting method. When there was no closed loop in the evidence network, the consistency model was directly used for analysis.

The pairwise comparison results between interventions were displayed in a league table, and each intervention was ranked by the surface under the cumulative ranking curve (SUCRA). In the range from 0 to 100, a higher SUCRA means a better intervention effect. Finally, funnel plot and Egger’s test were used to assess publication bias.

### 2.7 Quality of evidence

The evidence quality of NMA results was evaluated using an online platform CINeMA (Confidence in Network Meta-Analysis) (https://cinema.med.auth.gr). The credibility of the network evidence was evaluated from six aspects: within-study bias, reporting bias, indirectness, imprecision, heterogeneity and inconsistency. The quality of the evidence was divided into four levels: high, medium, low and very low.

### 2.8 Sensitivity analysis

The robustness of the NMA results for the primary outcome (serve velocity) was assessed via sensitivity analysis. Specifically, after two non-randomized controlled studies (Fernandez-Fernandez et al., 2016; Pardos-Mainer E et al., 2017) were excluded, the global inconsistency test and node-splitting analysis were repeated, and the SUCRAs and rankings were recalculated.

## 3 Results

### 3.1 Literature screening and research characteristics

**Figure 1:**
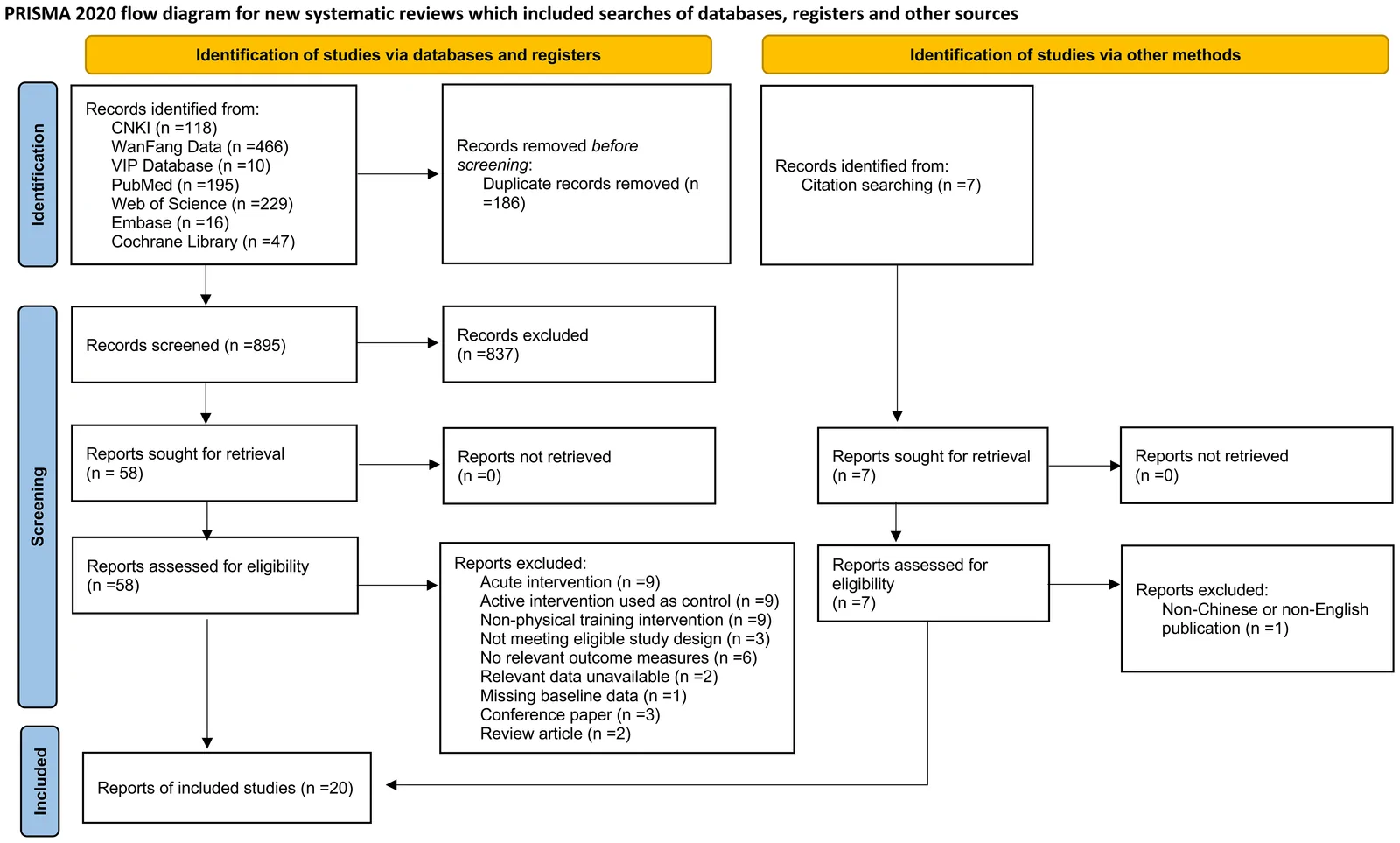
Literature screening flowchart.

Totally 1081 documents were obtained through database search. After the removal of 186 duplicate documents, the remaining 895 documents were screened according to the title and abstract. After the exclusion of 837 irrelevant studies, the remaining 58 documents were inputted into full-text assessment. Seven additional documents were retrieved and the full texts were evaluated by tracing the references of relevant systematic reviews. Finally, 20 studies were included (Behringer et al., 2013; Fernandez-Fernandez et al., 2013; Egesoy et al., 2021; Fernandez-Fernandez et al., 2016; Huangsheng and Zhongming, 2023; Pardos-Mainer E et al., 2017; Terraza-Rebollo et al., 2017; Treiber et al., 1998; Wang and Li, 2023; Wang and Xu, 2025; Guo, 2022, Mont et al., 1994; Malliou et al., 2011; Kara et al., 2015; Koçyiğit et al., 2020; Ölçücü et al., 2013; Liu, 2022; Baiget et al., 2023; Kraemer et al., 2000, 2003). The literature screening flow chart is shown in **Figure 1**.

This study involved totally 606 tennis players, including 319 players in the test group and 287 players in the control group. The competitive levels of the players cover young tennis players, college tennis players and professional tennis players, among which teenagers account for the majority. Interventions mainly include RT (8 studies), PT (6 studies), MMT (5 studies), and CST (3 studies). The training period ranges from 4 weeks to 9 months, the training frequency is 2 to 3 times a week, and the duration of a single training session is 25–105 minutes. In terms of outcome measures, 20, 6 and 6 studies reported serve velocity, serve accuracy, and upper-body power respectively. In terms of study design, 18 studies adopted a two-arm parallel control design, and 2 studies employed a three-arm parallel control design. Specific study characteristics were listed in **Supplementary Table 2**.

### 3.2 ROB assessment of included studies

In terms of random sequence generation, 16 studies were rated as low risk, 2 studies were rated as high risk of bias because they did not use randomization, and 2 studies were rated as unclear risk because the randomization method was not fully described. In terms of allocation scheme concealment, only 3 studies were rated as low risk, and the remaining 17 studies were rated as unclear risk because they did not provide sufficient allocation information. In terms of blinding of participants or interveners, it is difficult to implement blinding, due to the particularity of exercise training intervention. Nineteen studies were classified as high risk and only one study was classified as low risk. Regarding blinding of outcome evaluators, only one study was classified as low risk and the remaining 19 studies were classified as unclear risk. In terms of completeness of outcome data, all studies were at low risk of bias, except for one study that was lost to follow-up without explaining the reason. All studies were considered low risk in terms of selective reporting. In terms of other biases, 3 studies were at high risk and the remaining 17 studies were at low risk. Overall, the methodological quality of the included studies was moderate. The results of the ROB assessment of the included studies are detailed in **Supplementary Figures S1 and S2**.

### 3.3 Data analysis: main outcome indicators

#### 3.3.1 Serve velocity

**Figure 2:**
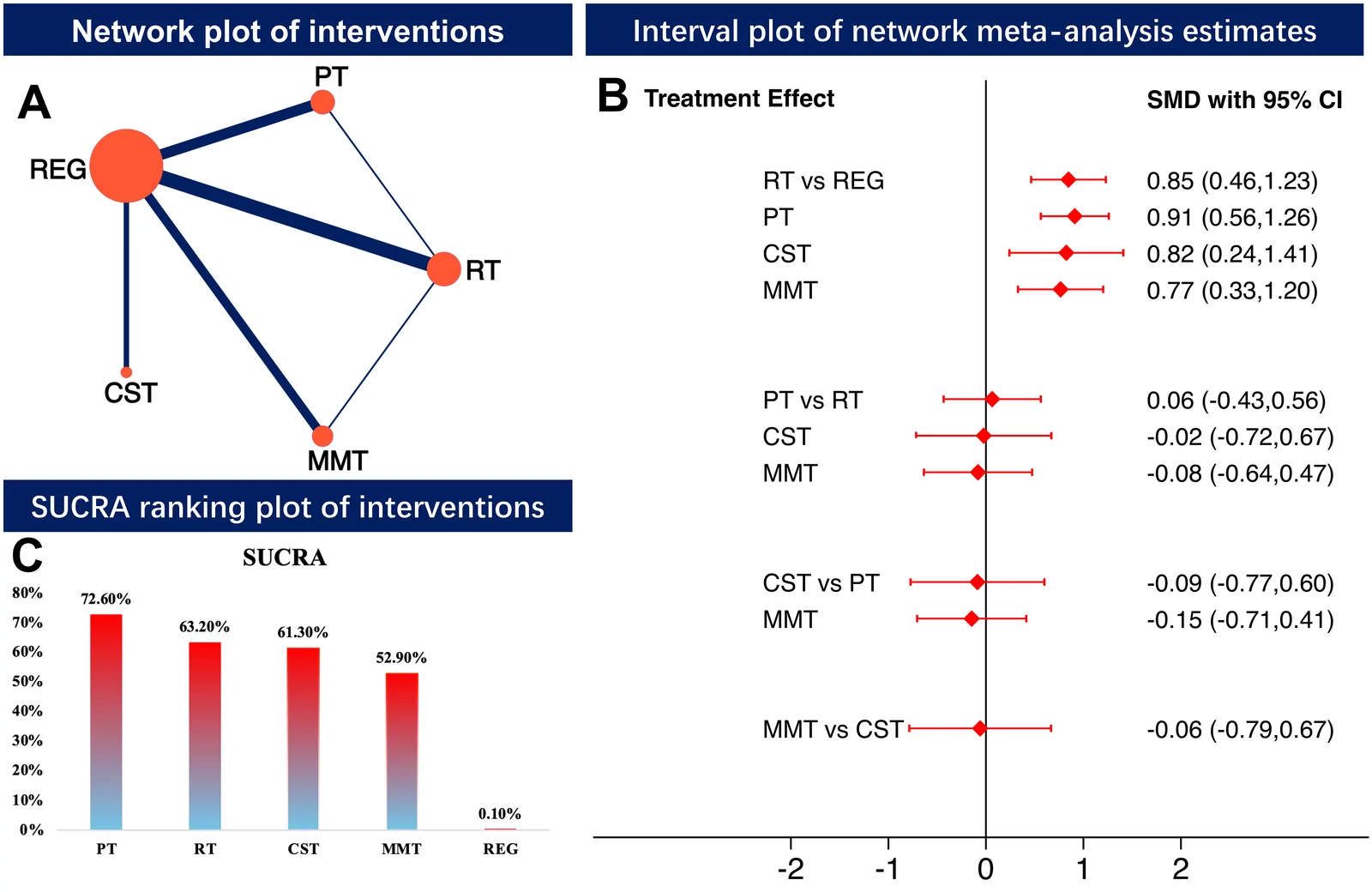
Network meta-analysis results for effects of different exercise training interventions on serve velocity. **(A)** Network plot of interventions; **(B)** interval plot of network meta-analysis estimates; **(C)** SUCRA ranking of interventions. In the network plot, node size represents the number of participants receiving each intervention, and line thickness represents the number of studies directly comparing two interventions. REG, regular training; RT, resistance training; PT, plyometric training; CST, core stability training; MMT, multimodal training; SUCRA, surface under the cumulative ranking curve; SMD, standardized mean difference; CI, confidence interval. (the same below).

The NMA included 20 studies with 606 tennis players, and compared 5 interventions to form 2 closed evidence networks (**Figure 2A**). The global inconsistency test showed that the overall consistency of the network was good (χ²=1.51, df=4, P = 0.83; **Supplementary Table 3**), so the consistency model was used for analysis. The test results of the node splitting method showed good consistency between the direct evidence and indirect evidence of each comparison (all P > 0.05), but found no significant local inconsistency (**Supplementary Table 4**).

The NMA showed that PT (SMD = 0.91, 95%CI: 0.56 to 1.26), RT (SMD = 0.85, 95%CI: 0.46 to 1.23), CST (SMD = 0.82, 95%CI: 0.24 to 1.41) and MMT (SMD = 0.77, 95%CI: 0.33 to 1.20) all significantly improved serve velocity compared with REG. No significant differences were found in comparisons between different training interventions (**Figure 2B**). According to the SUCRA, PT was the best intervention to improve serve velocity (72.6%), followed by RT (63.2%) (**Figure 2C**; **Supplementary Figure S3**).

**Figure 3:**
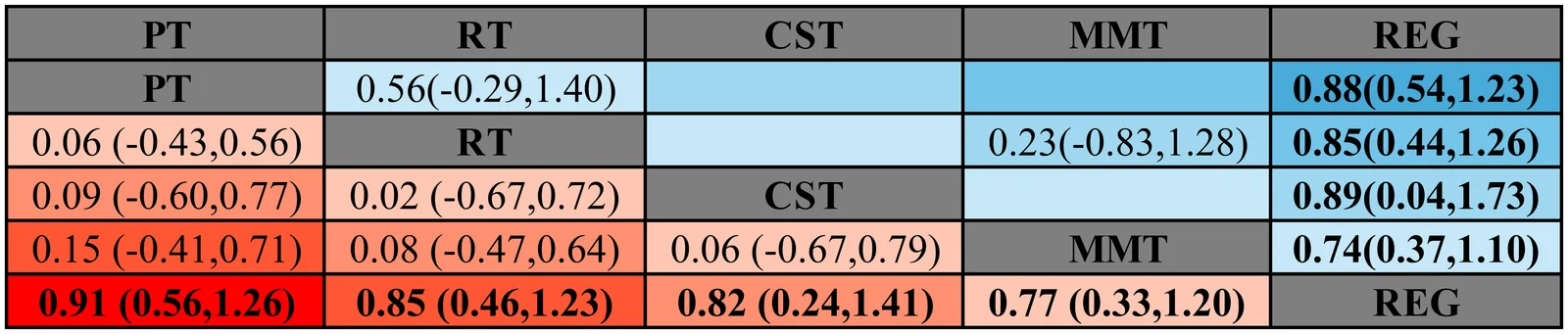
League table of pairwise and network meta-analysis results for serve velocity. Intervention effects are presented as SMDs with 95% CIs. For the network meta-analysis results (lower triangle), the column-defining intervention is favored; for the pairwise meta-analysis results (upper triangle), the row-defining intervention is favored. Bold values indicate statistically significant differences between interventions. (the same below).

The statistical significance levels of the PMA results and the NMA results are basically consistent (**Figure 3**). PT, RT, CST and MMT all significantly improved serve velocity compared with REG (95% CIs not crossing 0), while the differences between different specialized training interventions were not significant. Overall, all types of special training interventions are better than regular training, but there is no clear advantage or disadvantage between specialized training interventions.

#### 3.3.2 Serve accuracy

**Figure 4:**
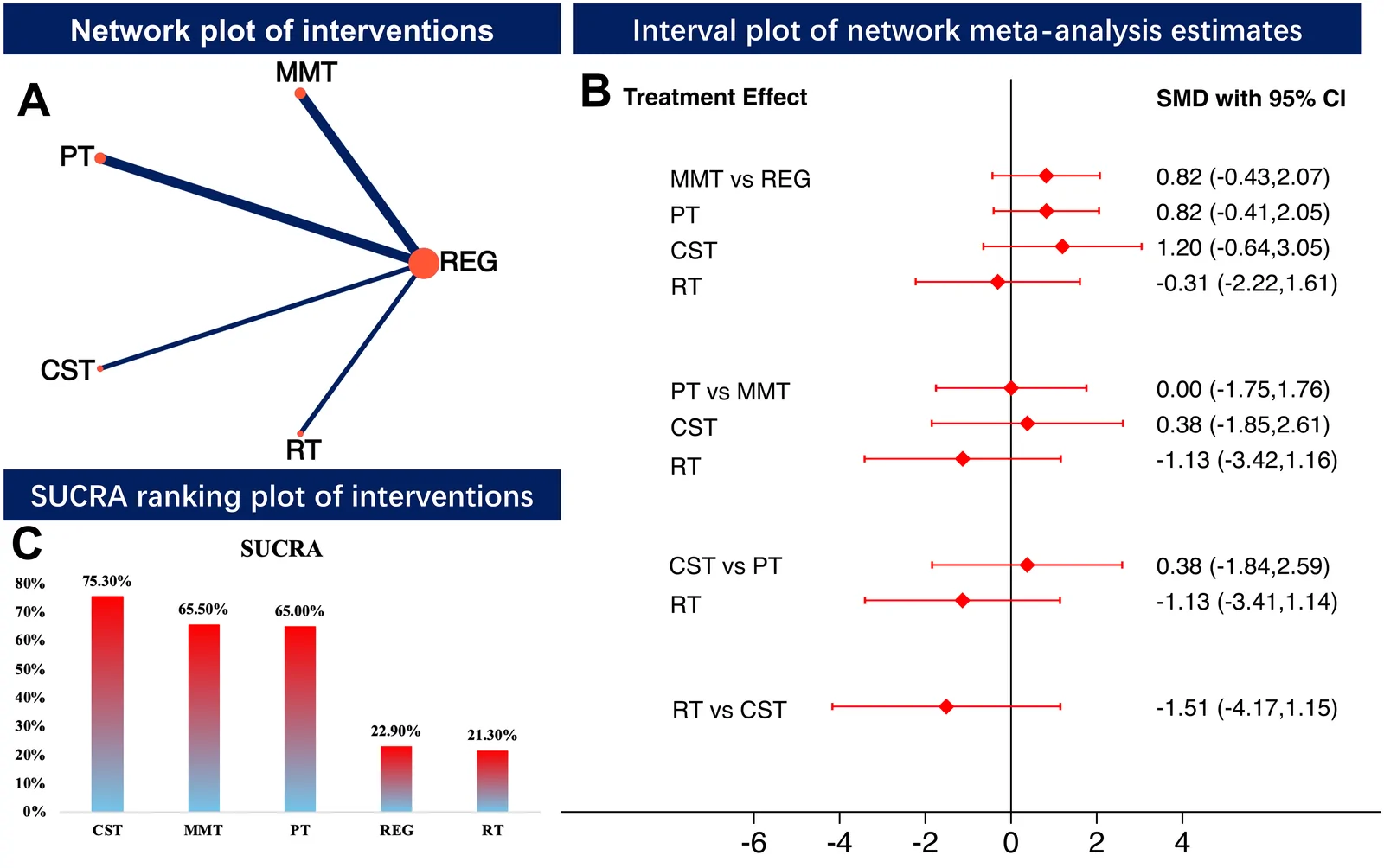
Network meta-analysis results for the effects of different exercise training interventions on serve accuracy. **(A)** Network plot of interventions; **(B)** interval plot of network meta-analysis estimates; **(C)** SUCRA ranking plot of interventions. In the network plot, node size represents the number of participants receiving each intervention, and line thickness represents the number of studies directly comparing two interventions.

NMA included 6 studies with 201 tennis players, compared 5 types of interventions, but did not form a closed evidence network (**Figure 4A**), and used the consistency model for analysis.

The NMA results showed that MMT (SMD = 0.82, 95%CI: -0.43 to 2.07), PT (SMD = 0.82, 95%CI: -0.41 to 2.05), CST (SMD = 1.20, 95%CI: -0.64 to 3.05) and RT (SMD = -0.31, 95%CI: -2.22 to 1.61) did not affect serve accuracy significantly compared with REG. Comparisons between specialized training interventions also found no significant differences, with all 95% CIs spanning 0 (**Figure 4B**). According to the SUCRA, CST ranks the highest (75.3%) and is the best training method to improve serve accuracy, while RT ranks the lowest (21.3%) (**Figure 4C**; **Supplementary Figure S4**). However, the CST and RT nodes were estimated based on a single study and the volume of evidence was limited, so the relevant results should be interpreted with caution.

**Figure 5:**
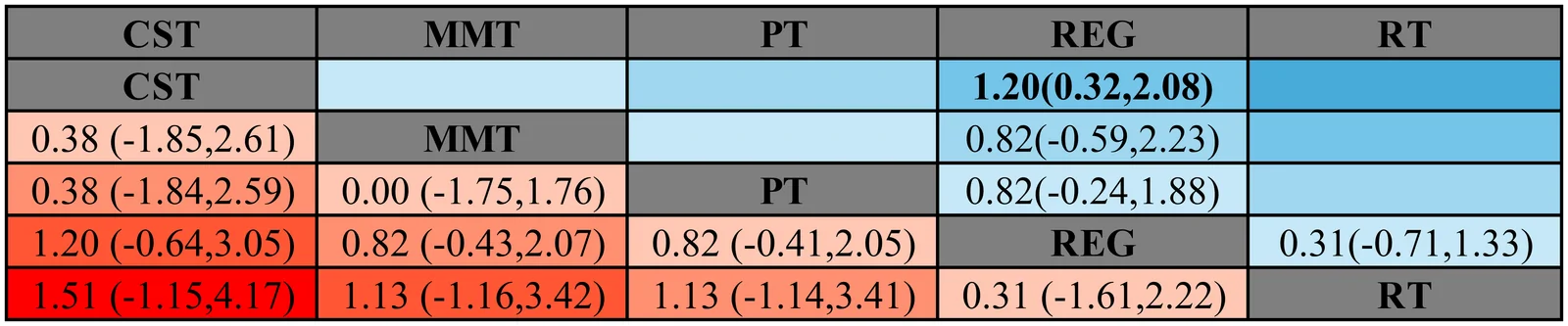
League table of pairwise and network meta-analysis results for serve accuracy. Intervention effects are presented as SMDs with 95% CIs.

The PMA results are not completely consistent with the NMA results in terms of significance (**Figure 5**). PMA showed that CST significantly improved serve accuracy compared with REG (SMD = 1.20, 95%CI: 0.32 to 2.08), while this comparison did not reach significance in NMA. The remaining comparison results are consistent with NMA and do not show significant differences. Overall, the PMA suggests that CST may positively affect serve accuracy. However, this conclusion needs to be interpreted with caution, due to limited evidence related to CST.

### 3.4 Data analysis: secondary outcome indicators

#### 3.4.1 Upper-body power

**Figure 6:**
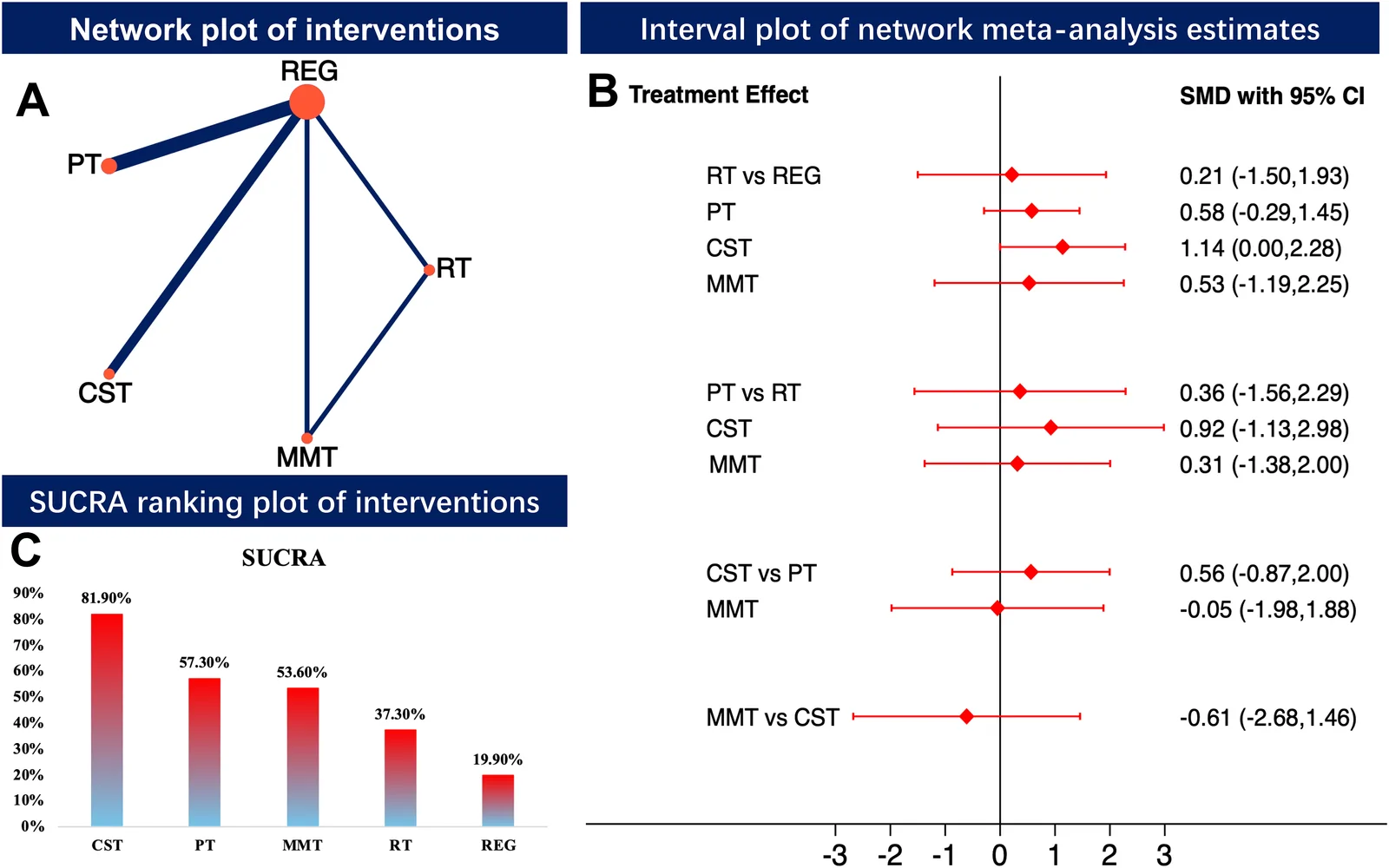
Network meta-analysis results for the effects of different exercise training interventions on upper-body power. **(A)** network plot of interventions; **(B)** interval plot of network meta-analysis estimates; **(C)** SUCRA ranking plot of interventions.

The NMA included 6 studies with 183 tennis players, and compared 5 interventions, forming a closed evidence network (**Figure 6A**). This closed loop was formed by the same three-arm study, so there was no need to conduct an inconsistency test, and a consistency model was used for analysis.

The NMA results showed that the effects of RT (SMD = 0.21, 95%CI: -1.50 to 1.93), PT (SMD = 0.58, 95%CI: -0.29 to 1.45) and MMT (SMD = 0.53, 95%CI: -1.19 to 2.25) on upper-body power did not reach significance compared with REG. CST showed a large positive effect, but the lower limit of its 95%CI was close to 0 (SMD = 1.14, 95%CI: 0.00 to 2.28). This result needs to be interpreted with caution. No significant differences were found in comparisons between specialized training, and all 95%CIs spanned 0 (**Figure 6B**). However, both RT and MMT nodes were estimated based on a single study, and the amount of evidence was limited, so the relevant results should be interpreted with caution. According to the SUCRA, CST is the best training method to improve the tennis players’ upper-body power (81.90%), followed by PT (57.30%) (**Figure 6C**; **Supplementary Figure S5**).

**Figure 7:**
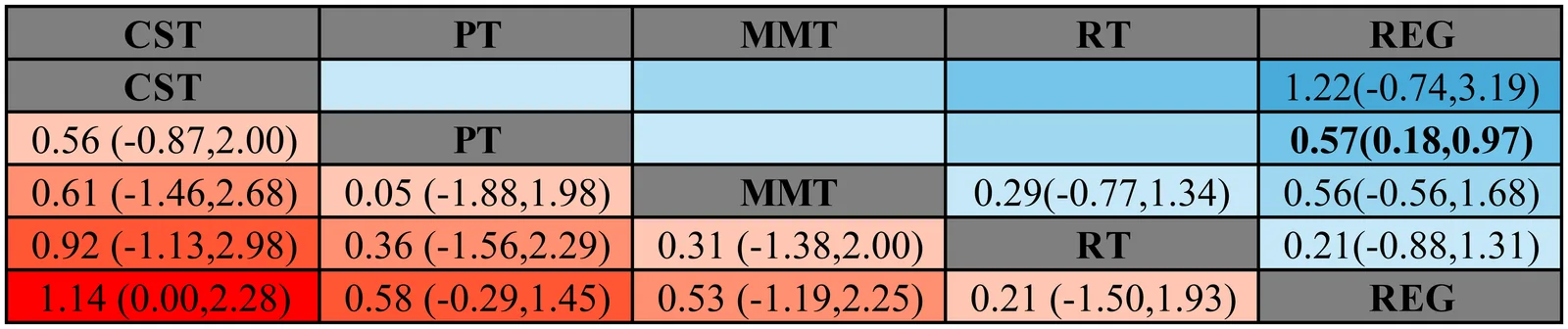
League table of pairwise and network meta-analysis results for upper-body power. Intervention effects are presented as SMDs with 95% CIs.

The PMA results are not completely consistent with the NMA results in terms of statistical significance (**Figure 7**). PMA showed that PT significantly improved upper-body power compared with REG (SMD = 0.57, 95%CI: 0.18 to 0.97), while this comparison did not reach statistical significance in NMA. On the contrary, CST showed a borderline positive effect compared with REG in NMA, but this comparison did not reach statistical significance in the PMA (SMD = 1.22, 95% CI: -0.74 to 3.19). The remaining comparison results are consistent with NMA and do not show significant differences. Overall, CST and PT may positively affect the upper-body power of tennis players.

### 3.5 Publication bias test

Funnel plots and Egger’s test were used to evaluate publication bias and the small sample effect on three outcome indicators: serve velocity, serve accuracy, and upper-body power. The funnel plots of the three indicators showed no obvious asymmetry (**Supplementary Figures S6–S8**). Egger’s test showed no significant publication bias in serve velocity (P = 0.777), serve accuracy (P = 0.997) or upper-body power (P = 0.550) (**Supplementary Tables S5–7**). In all, no obvious evidence of publication bias was found in any of the three outcome indicators. However, the numbers of studies included in serve accuracy and upper-body power were small, and the statistical power of the funnel plot and Egger’s test was limited, so the results need to be interpreted with caution.

### 3.6 Assessment of evidence quality

According to CINeMA, the quality of evidence for each outcome ranged from high to very low. Some comparisons on serve velocity reached high or medium quality of evidence, while the quality of evidence on serve accuracy and upper-body power was generally low, with most comparisons being low or very low. The downgrading of evidence is mainly related to imprecision, within-study bias and heterogeneity. Details are shown in **Supplementary Table 8**.

### 3.7 Sensitivity analysis

After the two non-randomized controlled studies (Fernandez-Fernandez et al., 2016; Pardos-Mainer E et al., 2017) were excluded, the global inconsistency test showed no significant inconsistency in the serve velocity network (χ² = 1.05, df = 4, P = 0.9017; **Supplementary Table 9**). Therefore, the consistency model was used. Node-splitting analysis also showed no significant differences between direct and indirect evidence for any estimable comparison (all P > 0.05; **Supplementary Table 10**). The SUCRA ranking was PT (88.3%) > RT (59.1%) > CST (54.3%) > MMT (48.2%) > REG (0.1%) (**Supplementary Table 11**). This ranking order was unchanged from the primary analysis, indicating that the SUCRA ranking for serve velocity is robust.

## 4 Discussion

The effects of different sports training methods on tennis players’ serve performance and upper-body power were systematically compared through PMA and NMA. The overall results show that all types of specialized training can significantly improve serve velocity compared with REG. Among them, PT, RT and CST rank the top three by SUCRA, suggesting that these training methods may have more advantages in improving serve velocity. In contrast, the effects of different training methods on serve accuracy and upper-body power are unclear. PMA shows that CST can significantly improve serve accuracy, and PT can significantly improve upper-body power. However, in NMA, these comparisons did not reach statistical significance. According to the SUCRA ranking, the top three interventions for serve accuracy are CST (SUCRA = 75.30%), MMT (SUCRA = 65.50%) and PT (SUCRA = 65.00%). The top three interventions for upper-body power are CST (SUCRA = 81.90%), PT (SUCRA = 57.30%) and MMT (SUCRA = 53.60%). However, these rankings reflect the probability of relative superiority rather than the effect size. Meanwhile, the rankings of serve accuracy and upper-body power should be interpreted with caution, due to sparse evidence.

PT is the most effective intervention to improve serve velocity, which has also been confirmed in previous studies on PT. A meta-analysis of 8 studies shows that PT has a moderate positive effect on tennis players’ serve velocity (effect size = 0.75), but there is no definite evidence that PT can improve serve accuracy, upper-body power or other indicators (Deng et al., 2022). An experimental study also shows that 8 weeks of PT can significantly increase the serve velocity of tennis players, but does not improve the serve accuracy (Behringer et al., 2013). Serve in tennis is a multi-joint action involving the whole body. The force generated by the lower limbs is gradually transferred to the shoulders, elbows, wrist and racket through the rotation of the hips and trunk, and finally completes the high-velocity hitting (Kovacs and Ellenbecker, 2011). Therefore, the improvement of serve velocity depends on not only the maximum strength level, but also the player’s ability to quickly generate force in very short time and efficiently transfer the force from the proximal link to the distal link (Kovacs and Ellenbecker, 2011; Colomar et al., 2024; Maffiuletti et al., 2016). Specifically, PT enhances rapid force output by improving the stretch-shortening cycle function and the neural drive and intermuscular coordination, and promotes force transfer efficiency in the service kinetic chain (Maffiuletti et al., 2016; Markovic and Mikulic, 2010). However, an increase in serve velocity does not mean a simultaneous increase in serve accuracy. The serve accuracy not only depends on the strength level, but is also affected by technical factors, such as the stability of throw, the height of the hitting point and the control of target placement (Mendes et al., 2013). In this study, PT only ranks third in terms of serve accuracy (SUCRA = 65.00%), indicating that the improving effect of PT on serve accuracy is relatively limited. Therefore, when using PT as a training method to improve serve velocity, it should be combined with other training (e.g., throwing training and placement control) to promote the overall improvement of serve performance.

In contrast, CST shows potential advantages in serve accuracy, which may be attributed to the improved stability of the trunk-pelvic region by CST. A stable core area provides proximal support for the upper limb hitting action, promotes the proximal to distal force transfer and coordination of movements, and thereby helps improve the consistency of serve and the ability to control placement (Kibler et al., 2006). Previous research also provides some support for CST to improve serve accuracy. Deng et al. (2023) found that physical training can significantly improve serve accuracy (ES = 1.14). However, this evidence only comes from 2 studies both on core stability training, suggesting that CST may positively affect serve accuracy, but the evidence base is relatively weak (Deng et al., 2023). The evidence supporting the effect of CST on serve accuracy in the present study remains limited. The serve accuracy network included only six studies, and the evidence for the CST node was derived from a single study. Furthermore, in the NMA, CST did not demonstrate a statistically significant advantage over REG or other training methods. Although CST ranked first according to SUCRA, suggesting that it may be the most promising training method for improving serve accuracy, this result should be regarded as exploratory and cannot support a definitive conclusion. Further validation in future studies is therefore required.

As for upper-body power, our results also need to be interpreted with caution. PMA shows that PT can significantly improve upper-body power compared with REG, but this comparison does not reach statistical significance in NMA, likely due to the sparse evidence network. Another meta-analysis shows that PT can improve medicine ball throwing performance (ES = 0.64) and special throwing performance (e.g., ball velocity in handball, baseball, and golf, ES = 0.55) (Garcia-Carrillo et al., 2023). Further tennis-specific research demonstrates that 8 weeks of PT could significantly improve tennis players’ medicine ball throwing and serve velocity (Wang and Xu, 2025). Explosive strength exercises in PT are similar in action patterns to medicine ball throwing test items, which may help improve upper limb quick strength. In addition, CST in this study ranks the first in the SUCRA ranking, suggesting that it may have a better intervention effect. Core stability contributes to the transmission and control of force in sports (Kibler et al., 2006). The performance of medicine ball throwing movements not only depends on upper limb strength, but is also affected by trunk stability and core rotation ability (Ikeda et al., 2009). CST may positively impact medicine ball throwing performance by improving the stability of the core area and promoting force transfer to the upper limbs. Reportedly, 8 weeks of static and dynamic core training can significantly improve the seated medicine ball throwing performance and serve velocity of young tennis players (p< 0.05), with a medium effect size (Egesoy et al., 2021). However, the existing evidence is not entirely consistent. Söğüt (2016) demonstrate no significant correlation between core stability and medicine ball throwing or serve performance in adolescent tennis players. However, in their study, the confidence interval of CST is wider than that of REG, and the amount of relevant evidence is limited. Therefore, although CST ranks the highest in terms of upper-body power, it is yet uncertain whether it is superior to other training methods, and this result can be regarded as an exploratory finding.

Although PMA and NMA differ in statistical significance for serve accuracy and upper-body power, they are highly consistent in the direction and point estimates of the effects. For serve accuracy, the effect estimate for CST versus REG is identical in PMA and NMA (SMD = 1.20), but the 95% CI is widened from 0.32 to 2.08 in PMA to −0.64 to 3.05 in NMA. Similarly, for upper-body power, the effect estimates for PT versus REG are comparable between PMA (SMD = 0.57) and NMA (SMD = 0.58), but the 95% CI is widened from 0.18 to 0.97 to −0.29 to 1.45. Therefore, these differences mainly reflect reduced estimation precision rather than contradictory intervention effects. The reason may be that only six studies were included for each outcome across five intervention nodes, resulting in sparse network evidence. In addition, the serve accuracy network contains no closed loop, whereas the closed loop in the upper-body power network originates from a single three-arm study; therefore, the network structure does not provide sufficient independent evidence for the relevant comparisons. When direct evidence is limited and the network structure is sparse, NMA does not necessarily achieve greater estimation precision than PMA. Moreover, the limited sample size and insufficient statistical power may have reduced the stability of the statistically significant findings in PMA. Therefore, although PMA suggests that CST may improve serve accuracy and that PT may improve upper-body power, the combined NMA findings and the existing evidence remain insufficient to draw definitive conclusions regarding these effects.

Practically, PT can be used as a priority training method to improve serve velocity, especially for tennis players who need to develop rapid strength. Given the relatively high proportion of youth players in the included studies, the current evidence is more directly applicable to young populations. For players of other ages and competitive levels, the findings may still inform training design. However, the advantage of PT for these players requires further investigation. Since the effect of PT on serve accuracy remains unclear, PT should not be used alone to improve overall serve performance but be combined with serve technique, toss stability, and placement control training. In terms of serve accuracy, CST may have potential value by improving core stability and movement consistency. However, the current evidence is insufficient to support CST as an independent method for improving serve placement control, and it may be more appropriately used as an adjunct to serve accuracy training.

## 5 Limitations

This study has limitations. First, although many included studies focus on serve velocity, the numbers of studies on serve accuracy and upper-body power are limited, and some intervention nodes only consist of a few studies, which may affect the stability of network estimation and SUCRA ranking. Second, the included studies differ in player age, competitive level, training experience, training period, training frequency and outcome measurement methods, which may increase heterogeneity. Third, although the included studies involved young, collegiate, and professional players, young players accounted for a relatively large proportion of the overall sample. Therefore, the applicability of the findings across players of different ages and competitive levels warrants further investigation. Fourth, some studies have methodological deficiencies in randomization, allocation concealment, and blinding, which may affect the quality of evidence. Finally, some studies do not fully report the standard deviation of the change value and we need to estimate it based on the correlation coefficient, which may bring certain uncertainty to the effect size estimate.

## 6 Conclusions

This study systematically compares the effects of different exercise training interventions on tennis players’ serve performance and upper-body power through pairwise meta-analysis and network meta-analysis. Compared with regular training, all types of special training interventions can significantly improve serve velocity, and plyometric training (SUCRA = 72.6%) and resistance training (SUCRA = 63.2%) rank the top two. In terms of serve accuracy and upper-body power, there is no significant difference between each training intervention and regular training. CST ranks the highest by SUCRA in both serve accuracy (75.3%) and upper-body power (81.9%). However, due to the limited amount of evidence, this result should be regarded as an exploratory finding. Future research needs to further verify the impact of different training methods on tennis players’ serve accuracy and upper-body power through large-sample high-quality randomized controlled trials, and explore the differences in training effects among people of different ages, genders and competitive levels.

## References

[B35] Baiget E. Colomar J. Corbi F. (2023). Six-week joint-specific isometric strength training improves serve velocity in young tennis players. Int. J. Sports Physiol. Perform. 18, 148–156. doi: 10.1123/ijspp.2022-0292 36586413

[B14] Behringer M. Neuerburg S. Matthews M. Mester J. (2013). Effects of two different resistance-training programs on mean tennis-serve velocity in adolescents. Pediatr. Exerc Sci. 25, 370–384. doi: 10.1123/pes.25.3.370 23986524

[B2] Bonato M. Maggioni M. A. Rossi C. Rampichini S. La Torre A. Merati G. (2015). Relationship between anthropometric or functional characteristics and maximal serve velocity in professional tennis players. J. Sports Med. Phys. Fitness 55, 1157–1165. 24998615

[B1] Cavedon V. Zancanaro C. Milanese C. (2014). Kinematic analysis of the wheelchair tennis serve: Implications for classification. Scand. J. Med. Sci. Sports 24, e381–e388. doi: 10.1111/sms.12182 25371933

[B18] Chi K. Y. Li M. Y. Chen C. Kang E. (2023). Ten circumstances and solutions for finding the sample mean and standard deviation for meta-analysis. Syst. Rev. 12, 62. doi: 10.1186/s13643-023-02217-1 37005690 PMC10068165

[B40] Colomar J. Corbi F. Baiget E. (2024). Sex-related differences in physical determinants of young high-performance tennis players' serve velocity. Apunt Educ. Fís Esports. 2024, 58–67. doi: 10.5672/apunts.2014-0983.es.(2024/3).157.07

[B6] Colomar J. Corbi F. Brich Q. Baiget E. (2022). Determinant physical factors of tennis serve velocity: A brief review. Int. J. Sports Physiol. Perform. 17, 1159–1169. doi: 10.1123/ijspp.2022-0091 35894981

[B45] Deng N. Soh K. G. Abdullah B. Huang D. Sun H. Xiao W. (2023). Effects of physical training programs on female tennis players’ performance: a systematic review and meta-analysis. Front. Physiol. 14, 1234114. doi: 10.3389/fphys.2023.1234114 37664429 PMC10470022

[B38] Deng N. Soh K. G. Huang D. Abdullah B. Luo S. Rattanakoses W. (2022). Effects of plyometric training on skill and physical performance in healthy tennis players: A systematic review and meta-analysis. Front. Physiol. 13, 1024418. doi: 10.3389/fphys.2022.1024418 36505069 PMC9729950

[B17] Deng N. Soh K. G. Xu F. Yang X. (2024). The effects of strength and conditioning interventions on serve speed in tennis players: a systematic review and meta-analysis. Front. Physiol. 15, 1469965. doi: 10.3389/fphys.2024.1469965 39839526 PMC11747802

[B20] Egesoy H. Oksuzoglu A. Y. Ilhan A. (2021). The effect of static and dynamic core training on some motoric charactristic and tennis service velocity of tennis athletes. Int. J. Life. Pharma Res. 2021, 303–314.

[B15] Fernandez-Fernandez J. Ellenbecker T. Sanz-Rivas D. Ulbricht A. Ferrautia A. (2013). Effects of a 6-week junior tennis conditioning program on service velocity. J. Sports Sci. Med. 12, 232–239. 24149801 PMC3761833

[B21] Fernandez-Fernandez J. Saez de Villarreal E. Sanz-Rivas D. Moya M. (2016). The effects of 8-week plyometric training on physical performance in young tennis players. Pediatr. Exerc Sci. 28, 77–86. doi: 10.1123/pes.2015-0019 26252503

[B3] Fitzpatrick A. Stone J. A. Choppin S. John K. (2024). How are elite tennis matches won at Wimbledon? A comparison of close and one‐sided contests. Eur. J. Sport Sci. 24, 190–199. doi: 10.1002/ejsc.12063 41531421

[B46] Garcia-Carrillo E. Ramirez-Campillo R. Thapa R. K. Afonso J. Granacher U. Izquierdo M. (2023). Effects of upper-body plyometric training on physical fitness in healthy youth and young adult participants: a systematic review with meta-analysis. Sports Med. - Open 9, 93. doi: 10.1186/s40798-023-00631-2 37833510 PMC10575843

[B9] Groppel J. L. Roetert E. P. (1992). Applied physiology of tennis. Sports Med. 14, 260–268. doi: 10.2165/00007256-199214040-00004 1475554

[B11] Guo Y. Xie J. Dong G. Bao D. (2024). A comprehensive review of training methods for physical demands in adolescent tennis players: a systematic review. Front. Physiol. 15, 1449149. doi: 10.3389/fphys.2024.1449149 39345786 PMC11427912

[B28] Guo Z. (2022). Experimental study on the influence of core strength training on tennis players’ serving ability. Liaoning Sport Sci. Technol. 44, 137–140. doi: 10.13940/j.cnki.lntykj.2022.03.022

[B22] Huangsheng K. Zhongming X. (2023). Impacts of medicine ball training on explosive strength in tennis players’ upper bodies. Rev. Bras. Med. Esporte. 29, e2023_0013. doi: 10.1590/1517-8692202329012023_0013

[B47] Ikeda Y. Miyatsuji K. Kawabata K. Fuchimoto T. Ito A. (2009). Analysis of trunk muscle activity in the side medicine-ball throw. J. Strength Cond Res. 23, 2231–2240. doi: 10.1519/JSC.0b013e3181b8676f 19826303

[B31] Kara E. Aksıt T. Ozkol M. Z. Isik T. (2015). Effects of 6 week tennis specific exercises program on service velocity. Turk. J. Sport Exerc. 17, 71–76. doi: 10.15314/tjse.05384

[B7] Keller M. Kuhn Y. A. Lüthy F. Taube W. (2021). How to serve faster in tennis: The influence of an altered focus of attention and augmented feedback on service speed in elite players. J. Strength Cond Res. 35, 1119–1126. doi: 10.1519/jsc.0000000000002899 30531414

[B44] Kibler W. B. Press J. Sciascia A. (2006). The role of core stability in athletic function. Sports Med. 36, 189–198. doi: 10.2165/00007256-200636030-00001 16526831

[B32] Koçyiğit B. Akin S. Şentürk A. (2020). The effects of combined trainings on tennis serve speed in tennis players. Turkiye Klinikleri J. Sports Sci. 12, 137–146. doi: 10.5336/sportsci.2019-70168

[B13] Kovacs M. S. (2006). Applied physiology of tennis performance. Br. J. Sports Med. 40, 381–385. doi: 10.1136/bjsm.2005.023309 16632565 PMC2653871

[B39] Kovacs M. Ellenbecker T. (2011). An 8-stage model for evaluating the tennis serve: implications for performance enhancement and injury prevention. Sports Health 3, 504–513. doi: 10.1177/1941738111414175 23016050 PMC3445225

[B37] Kraemer W. J. Hakkinen K. Triplett-Mcbride N. T. Fry A. C. Koziris L. P. Ratamess N. A. . (2003). Physiological changes with periodized resistance training in women tennis players. Med. Sci. Sports Exerc. 35, 157–168. doi: 10.1097/00005768-200301000-00024 12544650

[B36] Kraemer W. J. Ratamess N. Fry A. C. Triplett-McBride T. Koziris L. P. Bauer J. A. . (2000). Influence of resistance training volume and periodization on physiological and performance adaptations in collegiate women tennis players. Am. J. Sports Med. 28, 626–633. doi: 10.1177/03635465000280050201 11032216

[B16] Lambrich J. Muehlbauer T. (2022). Effects of athletic training on physical fitness and stroke velocity in healthy youth and adult tennis players: A systematic review and meta-analysis. Front. Sports Act. Living. 4, 1061087. doi: 10.3389/fspor.2022.1061087 36704261 PMC9872018

[B34] Liu X. (2022). Methods of core strength training in college tennis players. Rev. Bras. Med. Esporte. 28, 771–774. doi: 10.1590/1517-8692202228062022_0103

[B41] Maffiuletti N. A. Aagaard P. Blazevich A. J. Folland J. Tillin N. Duchateau J. (2016). Rate of force development: physiological and methodological considerations. Eur. J. Appl. Physiol. 116, 1091–1116. doi: 10.1007/s00421-016-3346-6 26941023 PMC4875063

[B30] Malliou P. Papadimitriou D. Malliou V. Beneka A. Pafis G. Katsikas C. . (2011). The effect of strength training on tennis service performance of junior tennis players. Exerc Qual. Life. 3, 31–40. doi: 10.31382/EQOL201101057M

[B8] Maquirriain J. Baglione R. Cardey M. (2016). Male professional tennis players maintain constant serve speed and accuracy over long matches on grass courts. Eur. J. Sport Sci. 16, 845–849. doi: 10.1080/17461391.2016.1156163 26960753

[B42] Markovic G. Mikulic P. (2010). Neuro-musculoskeletal and performance adaptations to lower-extremity plyometric training. Sports Med. 40, 859–895. doi: 10.2165/11318370-000000000-00000 20836583

[B43] Mendes P. C. Fuentes J. P. Mendes R. Martins F. M. Clemente F. M. Couceiro M. S. (2013). The variability of the serve toss in tennis under the influence of artificial crosswind. J. Sports Sci. Med. 12, 309–315. 24149810 PMC3761841

[B29] Mont M. A. Cohen D. B. Campbell K. R. Gravare K. Mathur S. K. (1994). Isokinetic concentric versus eccentric training of shoulder rotators with functional evaluation of performance enhancement in elite tennis players. Am. J. Sports Med. 22, 513–517. doi: 10.1177/036354659402200413 7943517

[B5] O’Donoghue G. P. Brown E. (2008). The importance of service in Grand Slam singles tennis. Int. J. Perform. Anal. Sport 8, 70–78. doi: 10.1080/24748668.2008.11868449 37339054

[B33] Ölçücü B. Erdil G. Altınkök M. (2013). Evaluation of the effect of plyometric exercises on the speed of the ball and the hitting percentage during a service. Beden Eğit Spor Bilim Derg. 7, 48–59.

[B23] Pardos-Mainer E. Ustero-Perez O. Gonzalo-Skok O. (2017). Effects of upper and lower body plyometric training on physical performance in young tennis players. RICYDE Rev. Int. Cienc. Deporte 13, 225–243. doi: 10.5232/ricyde2017.04903

[B4] Prieto-Lage I. Paramés-González A. Torres-Santos D. Argibay-González J. C. Reguera-López-de-la-Osa X. Gutiérrez-Santiago A. (2023). Match analysis and probability of winning a point in elite men’s singles tennis. PloS One 18, e0286076. doi: 10.1371/journal.pone.0286076 37768928 PMC10538650

[B10] Qin H. Qian S. Zhou Y. (2026). Comparing the effects of singles vs. doubles high-intensity on-court tennis training and regular high-intensity interval training on aerobic and anaerobic performance adaptations: a randomized, parallel-controlled study. J. Sports Sci. Med. 25, 159–171. doi: 10.52082/jssm.2026.159 41710431 PMC12912681

[B48] Söğüt M. (2016). The relations between core stability and tennis-related performance determinants. Biol. Exercise. 12, 35–44. doi: 10.4127/jbe.2016.0107

[B24] Terraza-Rebollo M. Baiget E. Corbi F. Planas A. A. (2017). Efectos del entrenamiento de fuerza en la velocidad de golpeo en tenistas jóvenes. Rev. Int. Med. Cienc. Ac. 17, 349–366. doi: 10.15366/rimcafd2017.66.009

[B25] Treiber F. A. Lott J. Duncan J. Slavens G. Davis H. (1998). Effects of Theraband and lightweight dumbbell training on shoulder rotation torque and serve performance in college tennis players. Am. J. Sports Med. 26, 510–515. doi: 10.1177/03635465980260040601 9689369

[B19] Veronique A. C. Neil A. (2013). Exercise training for blood pressure: a systematic review and meta-analysis. J. Am. Heart Assoc. 2, e004473. doi: 10.1161/jaha.112.004473 23525435 PMC3603230

[B26] Wang J. Li Y. (2023). Strength training method for tennis players. Rev. Bras. Med. Esporte. 29, e2022_0632. doi: 10.1590/1517-8692202329012022_0632

[B27] Wang J. Xu Q. (2025). Single-session upper limb plyometric training is as effective as two sessions for improving muscle strength, power, and serve velocity in male youth tennis players: a randomized parallel controlled study. Front. Psychol. 16, 1539739. doi: 10.3389/fpsyg.2025.1539739 39931292 PMC11808127

[B12] Zhou Y. Bai Y. Liang Y. Yang K. Yang Y. (2025). Effects of neuromuscular training on tennis players: a systematic review and meta-analysis. BMC Sports Sci. Med. Rehabil. 17, 172. doi: 10.1186/s13102-025-01219-x 40605097 PMC12219898

