# Supplementary material for: Comparative effects of exercise training on serve performance and upper-body power in tennis players: a systematic review and network meta-analysis
Source: Front Physiol. 2026 Aug 28;17:1920673. doi: 10.3389/fphys.2026.1920673 (PMC13563649; doi:10.3389/fphys.2026.1920673)
Supplement: Supplementary file 1 [file SupplementaryFile1.docx]

***Supplementary materials***

**Contents**

| **Supplementary Table S1.** Search strategies | 2 |
| --- | --- |
| **Supplementary Table S2.** Characteristics of the included studies | 6 |
| **Supplementary Table S3.** Results of the global inconsistency test for the serve velocity network | 9 |
| **Supplementary Table S4.** Results of node-splitting analysis for local inconsistency in the serve velocity network | 9 |
| **Supplementary Table S5.** Egger’s test for publication bias in serve velocity | 10 |
| **Supplementary Table S6.** Egger’s test for publication bias in serve accuracy | 10 |
| **Supplementary Table S7.** Egger’s test for publication bias in upper-body power | 10 |
| **Supplementary Table S8.** CINeMA ratings for all comparisons of network meta-analysis outcomes | 11 |
| **Supplementary Table S9.** Results of the global inconsistency test for the serve velocity network in the sensitivity analysis | 13 |
| **Supplementary Table S10.** Results of node-splitting analysis for local inconsistency in the serve velocity network in the sensitivity analysis | 13 |
| **Supplementary Table S11.** SUCRA ranking results for serve velocity in the sensitivity analysis | 13 |
| **Supplementary Fig. S1.** Risk of bias graph of included studies | 14 |
| **Supplementary Fig. S2.** Risk of bias summary of included studies | 14 |
| **Supplementary Fig. S3.** Cumulative ranking probability curves for serve velocity | 15 |
| **Supplementary Fig. S4.** Cumulative ranking probability curves for serve accuracy | 15 |
| **Supplementary Fig. S5.** Cumulative ranking probability curves for upper-body power | 16 |
| **Supplementary Fig. S6.** Funnel plot for serve velocity | 17 |
| **Supplementary Fig. S7.** Funnel plot for serve accuracy | 17 |
| **Supplementary Fig. S8.** Funnel plot for upper-body power | 18 |

**Supplementary Table S1. Search strategies**

**PubMed(n=195)**

(("Tennis "[Mesh] OR Tennis players OR elite tennis athletes OR professional tennis players OR junior tennis players OR college tennis players OR university tennis players) AND ("Exercise "[Mesh] OR Physical training OR resistance training OR core stability training OR core training OR complex training OR combined training OR plyometric training OR plyometrics OR explosive power training)) AND ("Serve Velocity "[Mesh] OR Serve speed OR serving velocity OR serving speed OR ball speed OR ball velocity OR tennis serve performance OR tennis serve OR upper extremity explosive strength OR upper body power OR upper limb power) AND("Randomized Controlled Trials as Topic"[Mesh] OR Randomized Controlled Study OR Effect OR Efficacy OR Therapeutic Effect OR Impact OR Influence OR Clinical Trials, Randomized OR Trials, Randomized Clinical OR Controlled Clinical Trials, Randomized)

**Web of Science (n=229)**

#1 (((((((TS=(Tennis)) OR TS=(Tennis players)) OR TS=(elite tennis athletes)) OR TS=(professional tennis players)) OR TS=(junior tennis players)) OR TS=(college tennis players)) OR TS=(university tennis players))

#2 ((((((((((TS=(Exercise)) OR TS=(Physical training)) OR TS=(resistance training)) OR TS=(core stability training)) OR TS=(core training)) OR TS=(complex training)) OR TS=(combined training)) OR TS=(plyometric training)) OR TS=(plyometrics)) OR TS=(explosive power training))

#3 ((((((((((TS=(Serve Velocity)) OR TS=(Serve speed)) OR TS=(serving velocity)) OR TS=(serving speed)) OR TS=( ball speed)) OR TS=( ball velocity)) OR TS=( tennis serve performance)) OR TS=( tennis serve)) OR TS=(upper extremity explosive strength)) OR TS=(upper body power)) OR TS=(upper limb power)

#4 ((((((((((TS=(Randomized Controlled Trials as Topic)) OR TS=( Influence)) OR TS=( Impact)) OR TS=( Therapeutic Effect)) OR TS=( Efficacy)) OR TS=( Effect)) OR TS=( Randomized Controlled Study)) OR TS=(Clinical Trials, Randomized)) OR TS=(Trials, Randomized Clinical)) OR TS=(Controlled Clinical Trials, Randomized))

#5 #1 AND #2 AND #3 AND #4

**Embase (n=16)**

#1 ' Tennis'/exp

#2 ' Tennis players':ab,ti OR ' elite tennis athletes':ab,ti OR ' professional tennis players':ab,ti OR ' junior tennis players':ab,ti OR ' college tennis players':ab,ti OR ' university tennis players ':ab,ti

#3 #1 OR #2

#4 'Exercise '/exp

#5 'Physical training':ab,ti OR 'resistance training':ab,ti OR 'core stability training':ab,ti OR 'core training':ab,ti OR 'complex training':ab,ti OR 'combined training':ab,ti OR 'plyometric training':ab,ti OR 'plyometrics':ab,ti OR 'explosive power training':ab,ti

#6 #4 OR #5

#7 'Serve Velocity'/exp

#8 'Serve speed':ab,ti OR 'serving velocity':ab,ti OR 'serving speed':ab,ti OR 'ball speed':ab,ti OR 'ball velocity':ab,ti OR 'tennis serve performance':ab,ti OR 'tennis serve':ab,ti OR 'upper extremity explosive strength':ab,ti OR 'upper body power':ab,ti OR 'upper limb power':ab,ti

#9 #7 or #8

#10 'Randomized Controlled Trials as Topic '/exp

#11 'Randomized Controlled Study ':ab,ti OR 'Effect ':ab,ti OR 'Efficacy':ab,ti OR 'Therapeutic Effect ':ab,ti OR 'Influence ':ab,ti OR ' Clinical Trials ':ab,ti OR 'clinical trials, randomized':ab,ti OR 'trials, randomized clinical':ab,ti OR 'controlled clinical trials, randomized':ab,ti

#12 #10 or #11

#13 #3 AND #6 AND #9 AND #12

**Cochrane library (n=47)**

#1 MeSH descriptor: [Tennis] explode all trees

#2 (Tennis players):ti,ab,kw OR (elite tennis athletes):ti,ab,kw OR (professional tennis players):ti,ab,kw OR (junior tennis players):ti,ab,kw OR (college tennis players):ti,ab,kw OR (university tennis players):ti,ab,kw

#3 #1 OR #2

#4 MeSH descriptor: [Exercise] explode all trees

#5 (Physical training):ti,ab,kw OR (resistance training):ti,ab,kw OR (core stability training):ti,ab,kw OR (core training):ti,ab,kw OR (complex training):ti,ab,kw OR (combined training):ti,ab,kw OR (plyometric training):ti,ab,kw OR (plyometrics):ti,ab,kw OR (explosive power training):ti,ab,kw

#6 #4 OR #5

#7 (Athletic Performance):ti,ab,kw

#8 (Serve speed):ti,ab,kw OR (serving velocity):ti,ab,kw OR (serving speed):ti,ab,kw OR (ball speed):ti,ab,kw OR (ball velocity):ti,ab,kw OR (tennis serve performance):ti,ab,kw OR (tennis serve):ti,ab,kw OR (upper extremity explosive strength):ti,ab,kw OR (upper body power):ti,ab,kw OR (upper limb power):ti,ab,kw

#9 #7 OR #8

#10 MeSH descriptor: [Randomized Controlled Trials as Topic] explode all trees

#11 (Randomized Controlled Study):ti,ab,kw OR (Effect):ti,ab,kw OR (Efficacy):ti,ab,kw OR (Therapeutic Effect):ti,ab,kw OR (Impact):ti,ab,kw OR (Influence):ti,ab,kw OR (Clinical Trials, Randomized):ti,ab,kw OR (Trials, Randomized Clinical):ti,ab,kw OR (Controlled Clinical Trials, Randomized):ti,ab,kw

#12 #10 OR #11

#13 #3 AND #6 AND #9 AND #12

**CNKI(n=118)**

（主题：网球运动员）OR（主题：网球选手）OR（主题：精英网球运动员）AND（主题：职业网球运动员）OR（主题：青少年网球运动员）OR（主题：大学生网球运动员）AND（主题：训练）OR（主题：体能训练）OR（主题：阻力训练）OR（主题：核心稳定训练）OR（主题：复合训练）OR（主题：增强式训练）OR（主题：爆发力训练）AND（主题：发球速度）OR（主题：发球速率）OR（主题：发球表现）OR（主题：网球发球）OR（主题：球速）OR（主题：上肢爆发力）OR（主题：上肢力量）OR（主题：上肢功率）AND（主题：随机对照试验）OR（主题：随机对照研究）OR（主题：效果）OR（主题：疗效）OR（主题：影响）

**Wanfang Data(n=466)**

题名或关键词:(网球运动 or 网球选手 or 精英网球运动员 or 职业网球运动员 or 青少年网球运动员 or 大学生网球运动员) and 题名或关键词:(训练 or 体能训练 or 阻力训练 or 核心稳定训练 or 复合训练 or 增强式训练 or 爆发力训练) and 题名或关键词:(发球速度 or 发球速率 or 发球表现 or 网球发球 or 球速 or 上肢爆发力 or 上肢力量 or 上肢功率) and 题名或关键词:(随机对照试验 or 随机对照研究 or RCT or 疗效 or 效果 or 影响)

**VIP Database(n=10)**

题名或关键词=网球运动员 OR 网球选手 OR 精英网球运动员 OR 职业网球运动员 OR 青少年网球运动员 OR 大学生网球运动员 AND 题名或关键词=训练 OR 体能训练 OR 阻力训练 OR 核心稳定训练 OR 复合训练 OR 增强式训练 OR 爆发力训练 AND 题名或关键词=发球速度 OR 发球速率 OR 发球表现 OR 网球发球 OR 球速 OR 上肢爆发力 OR 上肢力量 OR 上肢功率 AND 题名或关键词=随机对照试验 OR 随机对照研究 OR 效果 OR 疗效 OR 影响

**Supplementary Table S2. Characteristics of the included studies**

| **Study** | **Mean Age (years)** | **Sample Size** | **Experience/Level** | **Intervention** | **Intervention Duration (Experimental Group)** | **Outcomes** |
| --- | --- | --- | --- | --- | --- | --- |
| Behringer et al. (2013) | EG1: 15.5 ± 0.9  EG2: 15.1 ± 1.8  CG: 14.6 ± 1.8 | EG1: 10  EG2:13  CG: 10 | junior tennis players | EG1: Plyometric training + regular tennis training  EG2: Resistance training + regular tennis training  CG: Regular tennis training | 8 weeks;  2 sessions/week;  45 min/session | SV |
| Egesoy et al. (2021) | 11.75 ± 1.48 | EG: 24  CG: 12 | 5.10 ± 1.74 years (junior tennis players) | EG: Core training + regular tennis training  CG: Regular tennis training | 8 weeks;  2 sessions/week; 25–30 min/session | SV, MBT |
| Fernandez-Fernandez et al. (2013) | EG: 13.2 ± 0.6  CG: 13.2 ± 0.5 | EG: 15  CG: 15 | ≥3 years (junior tennis players) | EG: Multimodal training (core, resistance, and medicine ball training) + regular tennis training  CG: Regular tennis training | 6 weeks;  3 sessions/week; 60–70 min/session | SV, SA |
| Fernandez-Fernandez et al. (2016) | 12.5 ± 0.3 | EG: 24  CG: 27 | ≥3 years (junior tennis players) | EG: Plyometric training + regular tennis training  CG: Regular tennis training | 8 weeks; 2 sessions/week;  30–60 min/session | SV, SA, MBT |
| Huangsheng et al. (2023) | EG: 21.55 ± 1.96  CG: 21.29 ± 1.11 | EG: 50  CG: 50 | Professional tennis players | EG: Plyometric training + regular tennis training  CG: Regular tennis training | 12 weeks | SV |
| Pardos-Mainer et al. (2017) | 14.33 ± 1.77 | EG: 11  CG: 10 | ≥4 years (junior tennis players) | EG: Plyometric training + regular tennis training  CG: Regular tennis training + regular physical conditioning training | 8 weeks;  2 sessions/week; | SV, MBT |
| Terraza-Rebollo et al. (2017) | EG1: 16.0 ± 0.8  EG2: 14.9 ± 0.7  CG: 15.4 ± 0.9 | EG1: 7  EG2: 7  CG: 6 | >4 years (junior tennis players) | EG1: Multimodal training (medicine ball throw and resistance training) + technical and tactical tennis training  EG2: Resistance training + technical and tactical tennis training  CG: Technical and tactical tennis training | 8 weeks;  3 sessions/week; 45 min/session | SV, MBT |
| Treiber et al. (1998) | 21.2 (18–29) | EG: 11  CG: 11 | Collegiate tennis players | EG: Resistance training + regular tennis training  CG: Regular tennis training | 4 weeks;  3 sessions/week | SV |
| Wang et al. (2023) | EG: 21.77 ± 1.37  CG: 21.91 ± 1.08 | EG: 20  CG: 20 | Collegiate tennis players | EG: Multimodal training (resistance and core training) + regular training  CG: Regular training | 8 weeks;  3 sessions/week;  40 min/session | SV, SA |
| Wang et al. (2025) | EG: 15.6 ± 1.0  CG: 15.7 ± 0.9 | EG: 16  CG: 15 | 3.6 ± 0.8 years (junior tennis players) | EG: Plyometric training + regular tennis training  CG: Regular tennis training | 8 weeks;  2 sessions/week | SV, MBT |
| Guo et al. (2022) | EG: 25.72 ± 3.58  CG: 24.83 ± 3.62 | EG: 12  CG: 12 | >5 years (professional tennis players) | EG: Core stability training + regular training  CG: Regular training | 12 weeks;  3 sessions/week;  40 min/session | SV, SA, MBT |
| Mont et al. (1994) | 33 (18–42) | EG: 8  CG: 13 | Professional tennis players | EG: Resistance training  CG: No additional training | 6 weeks;  3 sessions/week | SV |
| Malliou et al. (2011) | EG: 14.2 ± 0.8  CG: 14.4 ± 0.7 | EG: 12  CG: 12 | ≥2 years (junior tennis players) | EG: Resistance training + regular tennis training  CG: Regular tennis training | 7 weeks;  3 sessions/week; 90 min/session | SV |
| Kara et al. (2015) | EG: 22.8 ± 1.6  CG: 18.0 ± 0.0 | EG: 10  CG: 10 | Professional tennis players | EG: Multimodal training (medicine ball, resistance, and core training) + regular tennis training  CG: Regular tennis training | 6 weeks;  3 sessions/week; 45–60 min/session | SV |
| Koçyiğit et al. (2020) | EG: 13.0 ± 0.74  CG: 12.58 ± 0.67 | EG: 12  CG: 12 | 6–7 years (junior tennis players) | EG: Multimodal training (resistance, core, physical conditioning, and medicine ball training) + regular tennis training  CG: Regular tennis training | 12 weeks;  3 sessions/week; 90 min/session | SV |
| Ölçücü et al. (2013) | EG: 22.1 ± 1.33  CG: 22.0 ± 1.45 | EG: 20  CG: 20 | Professional tennis players | EG: Plyometric training + regular tennis training  CG: Regular tennis training | 8 weeks;  3 sessions/week; 105 min/session | SV, SA |
| Liu (2022) | Not reported | EG: 10  CG: 10 | Collegiate tennis players | EG: Core training  CG: Regular physical conditioning training | 14 weeks;  3 sessions/week; 30 min/session | SV |
| Baiget et al. (2023) | EG: 15.6 ± 1.1  CG: 15.5 ± 0.6 | EG: 10  CG: 6 | ≥5 years (junior tennis players) | EG: Resistance training + regular technical-tactical training + regular physical conditioning training  CG: Regular technical-tactical training + regular physical conditioning training | 6 weeks;  2–3 sessions/week | SV, SA |
| Kraemer et al. (2000) | EG: 19.0 ± 0.9  CG: 19.8 ± 1.7 | EG: 8  CG: 8 | 7.8 ± 2.4 years (collegiate tennis players) | EG: Resistance training + regular tennis training  CG: Regular tennis training | 9 months;  2–3sessions/week; ≤90 min/session | SV |
| Kraemer et al. (2003) | EG: 19.2 ± 1.1  CG: 19.3 ± 1.6 | EG: 9  CG: 8 | 8.1 ± 3.5 years (collegiate tennis players) | EG: Resistance training + regular tennis training  CG: Regular tennis training | 9 months;  2–3 sessions/week | SV |

**Note:** EG, experimental group; CG, control group; SV, serve velocity; SA, serve accuracy; MBT, medicine ball throw.

**Supplementary Table S3. Results of the global inconsistency test for the serve velocity network**

| **Statistic** | **Value** |
| --- | --- |
| χ² | 1.51 |
| Degrees of freedom (df) | 4 |
| P value | 0.8251 |

**Note:** Global inconsistency was assessed using the design-by-treatment interaction model. No significant inconsistency was detected in the network (P > 0.05).

**Supplementary Table S4. Results of node-splitting analysis for local inconsistency in the serve velocity network**

| **Comparison** | **Direct Estimate** | **SE** | **Indirect Estimate** | **SE** | **Difference** | **SE** | **P value** | **τ** |
| --- | --- | --- | --- | --- | --- | --- | --- | --- |
| REG vs RT | 0.8606 | 0.2086 | 0.7259 | 0.7737 | 0.1347 | 0.8076 | 0.868 | 0.2999 |
| REG vs PT | 0.8899 | 0.1888 | 1.3939 | 0.9925 | -0.5041 | 1.0128 | 0.619 | 0.3030 |
| REG vs MMT | 0.7582 | 0.2331 | 0.9942 | 1.1585 | -0.2361 | 1.1844 | 0.842 | 0.2965 |
| RT vs PT | 0.4030 | 0.5193 | -0.0561 | 0.3035 | 0.4590 | 0.6024 | 0.446 | 0.3018 |
| RT vs MMT | -0.2384 | 0.6119 | -0.0375 | 0.3269 | -0.2009 | 0.6939 | 0.772 | 0.2949 |

**Abbreviations:** SE, standard error; τ, heterogeneity parameter; REG, regular training; RT, resistance training; PT, plyometric training; MMT, multimodal training.

**Note:** Local inconsistency was assessed using the node-splitting method. No statistically significant inconsistency was detected between direct and indirect evidence for any comparison (all P > 0.05).

**Supplementary Table S5. Egger’s test for publication bias in serve velocity**

| **Std_Eff** | **Coef.** | **Std. Err.** | **t** | **P>\|t\|** | **95% Conf. Interval** |
| --- | --- | --- | --- | --- | --- |
| slope | -0.9171104 | 0.384564 | -2.38 | 0.026 | -1.714647 to -0.1195735 |
| bias | 0.2707947 | 0.9430518 | 0.29 | 0.777 | -1.684975 to 2.226564 |

**Note:** Std_Eff, standardized effect. Egger’s test showed no significant publication bias or small-study effects for serve velocity (P = 0.777).

**Supplementary Table S6. Egger’s test for publication bias in serve accuracy**

| **Std_Eff** | **Coef.** | **Std. Err.** | **t** | **P>\|t\|** | **95% Conf. Interval** |
| --- | --- | --- | --- | --- | --- |
| slope | -0.728577 | 1.73366 | -0.42 | 0.696 | -5.541183 to 4.084008 |
| bias | 0.0214311 | 4.69991 | 0.00 | 0.997 | -13.02761 to 13.07047 |

**Note:** Std_Eff, standardized effect. Egger’s test showed no significant publication bias or small-study effects for serve accuracy (P = 0.997).

**Supplementary Table S7.** Egger’s test for publication bias in upper-body power

| **Std_Eff** | **Coef.** | **Std. Err.** | **t** | **P>\|t\|** | **95% Conf. Interval** |
| --- | --- | --- | --- | --- | --- |
| slope | -0.0973868 | 0.8162959 | -0.12 | 0.909 | -2.094791 to 1.900017 |
| bias | -1.23163 | 1.94622 | -0.63 | 0.550 | -5.993858 to 3.530599 |

**Note:** Std_Eff, standardized effect. Egger’s test showed no significant publication bias or small-study effects for upper-body power (P = 0.550).

**Supplementary Table S8.** CINeMA ratings for all comparisons of network meta-analysis outcomes

| **Comparison** | **Nature of evidence** | **Within-study bias** | **Reporting bias** | **Indirectness** | **Imprecision** | **Heterogeneity** | **Incoherence** | **Confidence rating** |
| --- | --- | --- | --- | --- | --- | --- | --- | --- |
| **Serve velocity** | | | | | | | | |
| **REG vs RT** | mixed | Some concerns | Low risk | No concerns | No concerns | No concerns | No concerns | **High** |
| **REG vs PT** | mixed | Some concerns | Low risk | No concerns | No concerns | No concerns | No concerns | **High** |
| **REG vs CST** | mixed | Some concerns | Low risk | No concerns | No concerns | Some concerns | No concerns | **Moderate** |
| **REG vs MMT** | mixed | Some concerns | Low risk | No concerns | No concerns | Some concerns | No concerns | **Moderate** |
| **RT vs PT** | mixed | Some concerns | Low risk | No concerns | Major concerns | No concerns | No concerns | **Low** |
| **RT vs MMT** | mixed | Some concerns | Low risk | No concerns | Some concerns | Some concerns | No concerns | **Moderate** |
| **RT vs CST** | indirect | Some concerns | Low risk | No concerns | Major concerns | No concerns | Some concerns | **Low** |
| **PT vs CST** | indirect | Some concerns | Low risk | No concerns | Major concerns | No concerns | Some concerns | **Low** |
| **PT vs MMT** | indirect | Some concerns | Low risk | No concerns | Some concerns | Some concerns | Some concerns | **Low** |
| **CST vs MMT** | indirect | Some concerns | Low risk | No concerns | Major concerns | No concerns | Some concerns | **Low** |
| **Serve accuracy** | | | | | | | | |
| **REG vs MMT** | mixed | Some concerns | Low risk | No concerns | Some concerns | Some concerns | Some concerns | **Low** |
| **REG vs PT** | mixed | Major concerns | Low risk | No concerns | Some concerns | Some concerns | Some concerns | **Low** |
| **REG vs CST** | mixed | Some concerns | Low risk | No concerns | Major concerns | No concerns | Some concerns | **Low** |
| **REG vs RT** | mixed | No concerns | Low risk | No concerns | Major concerns | No concerns | Some concerns | **Low** |
| **MMT vs PT** | indirect | Some concerns | Low risk | No concerns | Major concerns | No concerns | Some concerns | **Low** |
| **MMT vs CST** | indirect | Some concerns | Low risk | No concerns | Major concerns | No concerns | Some concerns | **Low** |
| **MMT vs RT** | indirect | Some concerns | Low risk | No concerns | Major concerns | No concerns | Some concerns | **Low** |
| **PT vs CST** | indirect | Some concerns | Low risk | No concerns | Major concerns | No concerns | Some concerns | **Low** |
| **PT vs RT** | indirect | Some concerns | Low risk | No concerns | Major concerns | No concerns | Some concerns | **Low** |
| **CST vs RT** | indirect | Some concerns | Low risk | No concerns | Major concerns | No concerns | Some concerns | **Low** |
| **Upper-body power** | | | | | | | | |
| **REG vs RT** | mixed | Some concerns | Low risk | Some concerns | Major concerns | No concerns | Some concerns | **Low** |
| **REG vs PT** | mixed | Major concerns | Low risk | Some concerns | Some concerns | Some concerns | Some concerns | **Very low** |
| **REG vs CST** | mixed | Some concerns | Low risk | Some concerns | No concerns | Major concerns | Some concerns | **Low** |
| **REG vs MMT** | mixed | Some concerns | Low risk | Some concerns | Major concerns | No concerns | Some concerns | **Low** |
| **RT vs MMT** | mixed | Some concerns | Low risk | Some concerns | Major concerns | No concerns | Some concerns | **Low** |
| **RT vs PT** | indirect | Some concerns | Low risk | Some concerns | Major concerns | No concerns | Some concerns | **Low** |
| **RT vs CST** | indirect | Some concerns | Low risk | Some concerns | Major concerns | No concerns | Some concerns | **Low** |
| **PT vs CST** | indirect | Some concerns | Low risk | Some concerns | Major concerns | No concerns | Some concerns | **Low** |
| **PT vs MMT** | indirect | Some concerns | Low risk | Some concerns | Major concerns | No concerns | Some concerns | **Low** |
| **CST vs MMT** | indirect | Some concerns | Low risk | Some concerns | Major concerns | No concerns | Some concerns | **Low** |

**Note:** This table presents the CINeMA ratings for all comparisons in the network meta-analysis. Comparison indicates the interventions being compared. Nature of evidence: mixed (combination of direct and indirect evidence) or indirect (indirect evidence only). Confidence rating: high, moderate, low, or very low. Downgrading indicates the CINeMA domains responsible for downgrading: within-study bias, reporting bias, indirectness, imprecision, heterogeneity, and incoherence. Green, yellow, and red cells represent no/low concerns, some concerns, and major concerns, respectively. CINeMA, Confidence in Network Meta-Analysis.

**Supplementary Table S9.** Results of the global inconsistency test for the serve velocity network in the sensitivity analysis

| **Statistic** | **Value** |
| --- | --- |
| χ² | 1.05 |
| Degrees of freedom (df) | 4 |
| P value | 0.9017 |

**Note:** Global inconsistency was assessed using the design-by-treatment interaction model after excluding the two non-randomized studies. No significant inconsistency was detected in the network (P > 0.05).

**Supplementary Table S10.** Results of node-splitting analysis for local inconsistency in the serve velocity network in the sensitivity analysis

| **Comparison** | **Direct Estimate** | **SE** | **Indirect Estimate** | **SE** | **Difference** | **SE** | **P value** | **τ** |
| --- | --- | --- | --- | --- | --- | --- | --- | --- |
| REG vs RT | 0.8522 | 0.1991 | 0.9800 | 0.7477 | −0.1279 | 0.7800 | 0.870 | 0.2428 |
| REG vs PT | 1.0905 | 0.2104 | 1.3685 | 0.9398 | −0.2780 | 0.9666 | 0.774 | 0.2509 |
| REG vs MMT | 0.7531 | 0.2184 | 1.0153 | 1.1096 | −0.2621 | 1.1330 | 0.817 | 0.2377 |
| RT vs PT | 0.4104 | 0.4930 | 0.1731 | 0.3197 | 0.2373 | 0.5873 | 0.686 | 0.2542 |
| RT vs MMT | −0.2388 | 0.5860 | −0.0581 | 0.3071 | −0.1807 | 0.6616 | 0.785 | 0.2364 |

**Abbreviations:** SE, standard error; τ, heterogeneity parameter; REG, regular training; RT, resistance training; PT, plyometric training; MMT, multimodal training.

**Note:** Local inconsistency was assessed using the node-splitting method after excluding the two non-randomized studies. No statistically significant inconsistency was detected between direct and indirect evidence for any estimable comparison (all P > 0.05).

**Supplementary Table S11.** SUCRA ranking results for serve velocity in the sensitivity analysis

| **Rank** | **Intervention** | **SUCRA** |
| --- | --- | --- |
| 1 | PT | 88.3 |
| 2 | RT | 59.1 |
| 3 | CST | 54.3 |
| 4 | MMT | 48.2 |
| 5 | REG | 0.1 |

**Abbreviations:** REG, regular training; RT, resistance training; PT, plyometric training; CST, core stability training; MMT, multimodal training; SUCRA, surface under the cumulative ranking curve.

**Supplementary Fig. S1. Risk of bias graph of included studies.**

**Supplementary Fig. S2. Risk of bias summary of included studies.** Green circles indicate low risk of bias, yellow circles indicate unclear risk of bias, and red circles indicate high risk of bias.

**Supplementary Fig. S3. Cumulative ranking probability curves for serve velocity.** REG, regular training; RT, resistance training; PT, plyometric training; CST, core stability training; MMT, multimodal training.

**Supplementary Fig. S4. Cumulative ranking probability curves for serve accuracy.** REG, regular training; RT, resistance training; PT, plyometric training; CST, core stability training; MMT, multimodal training.

**Supplementary Fig. S5. Cumulative ranking probability curves for upper-body power.** REG, regular training; RT, resistance training; PT, plyometric training; CST, core stability training; MMT, multimodal training.

**Supplementary Fig. S6. Funnel plot for serve velocity.** The funnel plot was used to assess publication bias and small-study effects for serve velocity. REG, regular training; RT, resistance training; PT, plyometric training; CST, core stability training; MMT, multimodal training.

**Supplementary Fig. S7. Funnel plot for serve accuracy.** The funnel plot was used to assess publication bias and small-study effects for serve accuracy. REG, regular training; RT, resistance training; PT, plyometric training; CST, core stability training; MMT, multimodal training.

**Supplementary Fig. S8. Funnel plot for upper-body power.** The funnel plot was used to assess publication bias and small-study effects for upper-body power. REG, regular training; RT, resistance training; PT, plyometric training; CST, core stability training; MMT, multimodal training.
